# The mechanism of simultaneous intake of Jujuboside A and B in the regulation of sleep at the hypothalamic level

**DOI:** 10.18632/aging.204995

**Published:** 2023-09-05

**Authors:** Wei Wang, Yi Wang, Hongyan Pei, Mingming Li, Aozhe Zhu, Rui Du, Gao Jun Peng

**Affiliations:** 1 College of Humanities and College of Home Economics, Jilin Agricultural University, Changchun 130118, China; 2 College of Food Science and Engineering, Jilin Agricultural University, Changchun 130118, China; 3 New Vita Bioengineering Tianjin Co., Ltd, Tianjin 301726, China; 4 College of Chinese Medicinal Materials, Jilin Agricultural University, Changchun 130118, China

**Keywords:** jujuboside, quantitative proteomics, bioinformatics, improvement sleep, blood-brain barrier

## Abstract

To study the effect of co-administration of Jujuboside A and B (Ju A+B) on sleep, healthy KM mice were given different doses of Ju A+B with a behavioral evaluation of sleep state. Serum levels of key neurotransmitters (5HT, DA, and NE) were measured. The hypothalamus of KM mice was analyzed for differential protein expression using TMT quantitative proteomics, and differential expression protein (DEP) bioinformatics analysis was used to explore potential mechanisms. The result shows that Ju A+B affects sleep by expressing the protein in the hypothalamus. Compared with the control group, the test group showed 10 up-regulated and 139 down-regulated DEPs. The key DEPs were found at the tight junction. Western blot showed reliable alteration of the key DEPs in proteomics. The result of interaction network analysis attributed the potential of Jujuboside to the changes in blood-brain barrier, which provided basic and theoretical data for the efficacy evaluation and mechanism of Jujuboside.

## INTRODUCTION

*Ziziphus jujube* kernel is the mature dried seed of *Ziziphus jujube* Mill. It has been used since ancient times as a drug that exhibits diverse effects, including an anticonvulsant [1]. In addition, jujuboside (Ju), the main pharmacological component of *Ziziphus jujube* kernel, can significantly improve sleep [2]. Jujuboside belongs to the tetracyclic triterpenoids and pentacyclic triterpenoid saponins, of which there are more than 50 types [3]. Among them, Jujuboside A (JuA) and Jujuboside B (JuB) are the focus of most studies because they are considered to have the most pharmacological value. Furthermore, sleep improvement is one of the most significant effects of these triterpenoids [4].

Both JuA and JuB have specific effects on improving sleep [5], and the effect of JuA was slightly higher. Therefore, there are more studies focused on JuA., and the effect of JuA is slightly higher than that of JuB. Consequently, more studies have focused on JuA than JuB. The intake of Ju can alter neurotransmitter levels, including GABA and 5-HT, in the brain [6, 7], to improve sleep quality. JuA can also be transformed into JuB in the intestine [8]. However, in addition to interfering with the metabolism of some saponins in the body, some components or secondary metabolites may have effects on the blood-brain barrier and even directly on the brain tissue [9]. It is unknown whether a similar result can be achieved with the intake of Ju. It is also unclear whether the simultaneous intake of JuA and JuB will have a synergistic effect on improving sleep.

*Ziziphus jujube* kernel is an important resource for traditional Chinese medicine and is also a valuable food resource. According to the Food Safety Law of the People’s Republic of China and the provisions of the Health and Family Planning Commission, *Ziziphus jujube* belongs to a typical homology of medicine and food (MFH) material. It is likely to become a valuable functional food material if the resources of *Ziziphus jujube* kernels can be properly developed and utilized.

In this study, healthy Kunming mice (KM mice) were used as model animals, and fed different doses of JuA and JuB without the intervention of other drugs (such as barbital sodium). Behavioral observations were then used to assess the sleep state of the test animals. Serum levels of key neurotransmitters (5-HT, DA, and NE) were measured; TMT quantitative proteomics was used to determine differences in hypothalamic protein expression between animals; and differences in protein expression and metabolic pathways were analyzed by bioinformatics. This study provides a prospective theoretical basis for determining whether Ju intake has a direct effect on brain metabolism.

## MATERIALS AND METHODS

### Test materials and animals

Jujuboside A (JuA, CAS: 55466-04-1, ≥98%) and Jujuboside B (JuB, CAS: 55466-05-2, ≥98%) were provided by Chengdu Gelipu Biotechnology Co. Ltd (Chengdu, China). Diazepam (DZP, CAS: 439-14-5) was bought from Northeast Pharmaceutical Group (Shenyang, China). Eight to twelve-week-old male Kunming mice were purchased from Beijing Vital River Laboratory Animal Technology Co. Ltd (Beijing, China). Specific Pathogen Free (SPF) animal laboratory and the place of the open-field test were provided by Changchun Wish Technology Co. Ltd (Changchun, China).

### The process of animal experiment

All mice were housed under a controlled condition in individual cages at 22±3° C and 60-70% relative humidity with a 12 h dark/light cycle in an SPF environment and were allowed free access to the food and sterile water. After one week of acclimation, 21 day feeding period was conducted (food and drink freely). All animals were divided into 11 groups (n=10) with intragastric administration: control group (equivalent normal saline), JuA low/middle/high-dose group (10/20/30 mg/kg/d JuA), JuB low/middle/high-dose group (10/20/30 mg/kg/d JuB), JuA+B low/middle/high-dose group (7/14/21 mg/kg/d JuA +3/6/9 mg/kg/d JuB), and DZP group (6 mg/kg/d DZP).

Logitech HD Pro Capture video recording system with a C920 pro camera was used to record the sleep condition of test animals. During observation, one test animal was regarded as a sleeping state if there is no action for 10 consecutive minutes; for one group test animal, sleep stability was determined according to the number of animals entering sleep within 1 hour. The sleep test of all animals was under the same conditions but separated from human activities and each other to prevent interference. The experimental animals were sacrificed on Day 21 after extracting the eyeball blood, meanwhile, the hypothalamus was removed and stored at -80° C.

### Determination of neurotransmitter level

5-hydroxytryptamine (5-HT), Dopamine (DA), and Norepinephrine (NE) were selected as the representatives to determine the effect of drug intake on the level of neurotransmitters. The serum of different test animals was obtained after the blood was centrifuged at 3000g for 10 min at 4° C. The contents of 5-HT, DA, and NE in the hypothalamus and the serum were quantitative with the ELISA kit protocol (No. ML001891, No. ML002024, No. ML063805; Shanghai Enzyme-linked Biotechnology Co., Ltd., Shanghai, China). The results are represented by ng/mL serum.

### TMT quantitative proteomics

### Total protein extraction

The protein extraction process is related to the methods used in previous studies with a minor optimization described as follows [10]: the samples were ground separately in liquid nitrogen, lysed with PASP lysis buffer before sonicated on ice for 5 min. The lysate was centrifuged at 12,000 g for 15 min at 4° C before the supernatant was reduced with 10 mM DTT for 1 h at 56° C. The supernatant was alkylated with bulk iodoacetamide for 1 h at RT from light before fully mixed with the excessive precooling acetone, kept at -20° C for more than 2 h, and then centrifuged at 12,000 g for 15 min at 4° C to collect the precipitate, which was lysed with lysis buffer after washing with 1 ml precooling acetone.

### TMT labeling of peptides

The process is related to the method used in the previous study with a minor optimization described as follows [11]: each sample was added by DB lysis buffer to 100 μL, mixed, and digest with trypsin and 100 mM TEAB buffer at 37° C for 4 h, and then digest overnight after mixed with trypsin and CaCl_2_. Adjusting the pH of the sample to less than 3.0 before centrifuging at 12,000 g for 5 min at RT. The C18 desalting column was used to elute the supernatant completely, and the eluent was collected before being freeze-dried. The lyophilized substance was mixed oscillatory with 100 μL of 0.1 M TEAB buffer and 41 μL of TMT labeling reagent at RT for 2 h before being stopped with 8% ammonia. All labeled samples were mixed in equal volumes, desalted, and lyophilized.

### Separation of fractions

The Rigol L3000 HPLC system with a C18 column (Waters BEH C18, 4.6×250 mm, 5 μm) was used to fractionate the separation as the method and conditions described by the previous study [12].

### LC-MS/MS analysis

An EASY-nLC™ 1200 UHPLC system with a Q Exactive™ HF-X mass spectrometer (Thermo Fisher, USA) and C18 Nano-Trap chromatography column (4.5 cm x 75 μm, 3 μm) in data-dependent acquisition (DDA) mode was used to LC-MS/MS detection, construct the transition library, and analyze the data. The method and conditions are related to the descriptions in the previous study [12].

### Western blot analyses

The lysating, quantification, SDS-PAGE, and transfer of the protein in hypothalamus samples were carried out according to the method of Wang et al. [13]. The primary antibodies were selected as follows: DLG1 (A8542, ABclonal Technology Co., Ltd., Wuhan, China), Afadin (55102-1-AP, Proteintech Group, Inc, Rosemont, IL, USA), ZO-1(55296-1-AP, Proteintech Group, Inc, USA), Tuba3 (17191-1-AP, Proteintech Group, Inc, USA), MUPP1 (A15344, ABclonal Technology Co., Ltd., Wuhan, China), Claudin11 (A12478, ABclonal Technology Co., Ltd., Wuhan, China) and PATJ (A12063, ABclonal Technology Co., Ltd., Wuhan, China), β-actin was used as a control. After the incubation and chemiluminescence, the expression of the proteins was analyzed by an iBright CL1000 imaging system (Invitrogen, Singapore).

### Data analysis

### The identification and quantitation of protein

Mus_musculus_uniprot_2021_7_15 (86544 sequences) database was used to search the resulting spectra by the search engines: Proteome Discoverer 2.4 (PD 2.4, Thermo Fisher, USA). The searched parameters are carried out according to the previous study [14]. The quantitation results were analyzed by T-test, *p* < 0.05, and |log_2_ Fold Change (FC)| > 0.25 were considered as a significant difference, and the proteins were defined as differentially expressed proteins (DE proteins, DEPs).

### The functional analysis of protein and DEP

Gene Ontology (GO) functional analysis was conducted using the InterPro scan program against the non-redundant protein database [15] and the databases of KEGG were used to analyze the protein pathway. DEPs were used for Volcanic map analysis, cluster heat map analysis, and enrichment analysis of GO and KEGG. The probable protein-protein interactions were predicted using the STRING-db server (http://string.embl.de/) [16].

### Statistical analysis

All biological experiments were set with at least six groups with three times intra-group parallel experiments at least. All the data were presented as means $\left(\bar{x}\right)$ ± standard deviation (SD). The amount of protein expression was determined according to the gray value, and statistical analysis was conducted using SPSS 25.0 (SPSS Inc, Chicago, IL, USA). *p* < 0.05 was considered to be statistically significant. GraphPad Prism 6 (GraphPad Software Inc., San Diego, CA, USA) was used to draw the graphs, etc.

## Results and Discussion

### Sleep status of test animals

**Figure 1:**
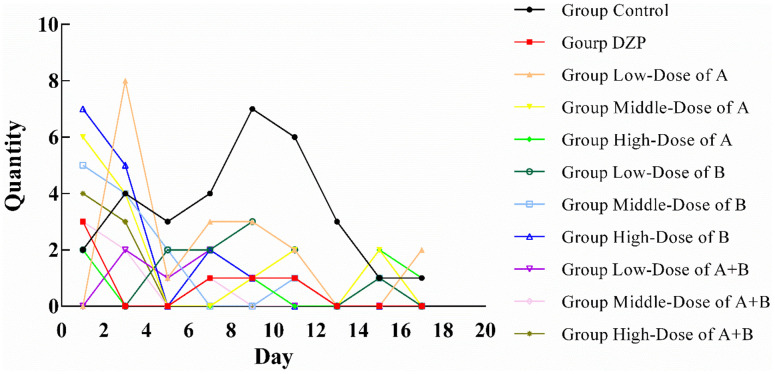
Number of test animals not sleeping in one hour (n=10).

The sleep status of all animals in this study is shown in Figure 1. All animals had poor sleep status at the first sleep observation, which was caused by the mice’s need to adapt to the new environment (the location of the open-field test). This situation improved significantly with the third observation. Compared with the control group, the sleep status of each test group improved with the extension of feeding time. The animals in Group DZP were able to enter a sleep state within 1 h from the 11th observation day, similar to those in Group JuA. The sleep status of the high-dose JuA group was slightly better than that of the medium and low-dose groups, in which the mice could fall asleep within 1 h from the 5th day of observation. One of them fell asleep within one hour in the latter observation, which might be caused by an external influence. JuB had a slightly better sleep-assisting effect than JuA. The mice in the low-dose group fell asleep within 1 h on the 13th day, whereas they fell asleep on the 7th and 5th days in the medium-dose and high-dose groups. However, possibly due to external interference, one animal did not fall asleep within 1 h. After the simultaneous intake of JuA and JuB, the sleep state of the test animals was significantly better than that of the other groups. At the low dose of JuA+B, all test animals fell asleep within 1 h on the 11th day, and in the medium and high dose groups, this occurred on the 9th day of the experiment. In addition, when JuA and JuB were ingested simultaneously after the animals fell asleep, the sleep condition was very stable in the follow-up observation, and there was no similar situation in the other groups.

In summary, the simultaneous intake of JuA and JuB had a better effect on assisted sleep, and the effect was better at high doses. Therefore, we chose the high-dose JuA+B and control groups for the follow-up proteomic experiment.

### The intake of JuA+B affects the level of neurotransmitters in the serum

**Figure 2:**
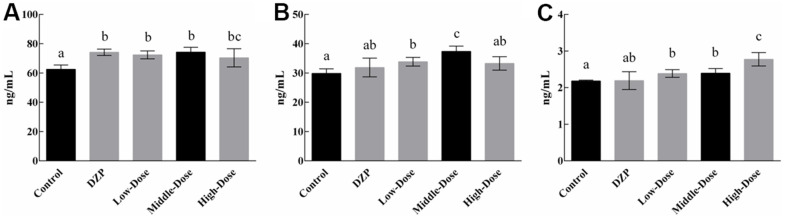
The content of 5-HT (**A**), DA (**B**), and NE (**C**) in the serum of the test animals Different lowercase letters represent significant differences (*p*<0.05).

The serum levels of the neurotransmitters are shown in Figure 2. Generally, the simultaneous intake of JuA+B exerted an impact on the expression levels of the three types of neurotransmitters. The level of 5-HT showed a slight increase with drug intake (Figure 2A), which was significantly higher than the control group (*p*<0.05), similar to the DZP group, and there was no dose dependence. The level of DA in the medium-dose group (Figure 2B) was significantly higher than that in the control and other groups (*p*<0.05), but lower than that in the high-dose group. The simultaneous intake of two types of saponins had a dose-dependent effect on NE levels (Figure 2C), and the NE level in the high-dose group was significantly higher than that in the control and other experimental groups (*p*<0.05). Since the tested animals did not show any differences during feeding other than drug intake, it is reasonable to conclude that the differential expression of neurotransmitters was caused by the intake of JuA + B. Although numerous studies have shown a strong relationship between the expression of these neurotransmitters and sleep status, it remains to be determined at the protein level what changes are produced in brain tissue.

### Protein expression differences of the hypothalamus induced by JuA+B

**Figure 3:**
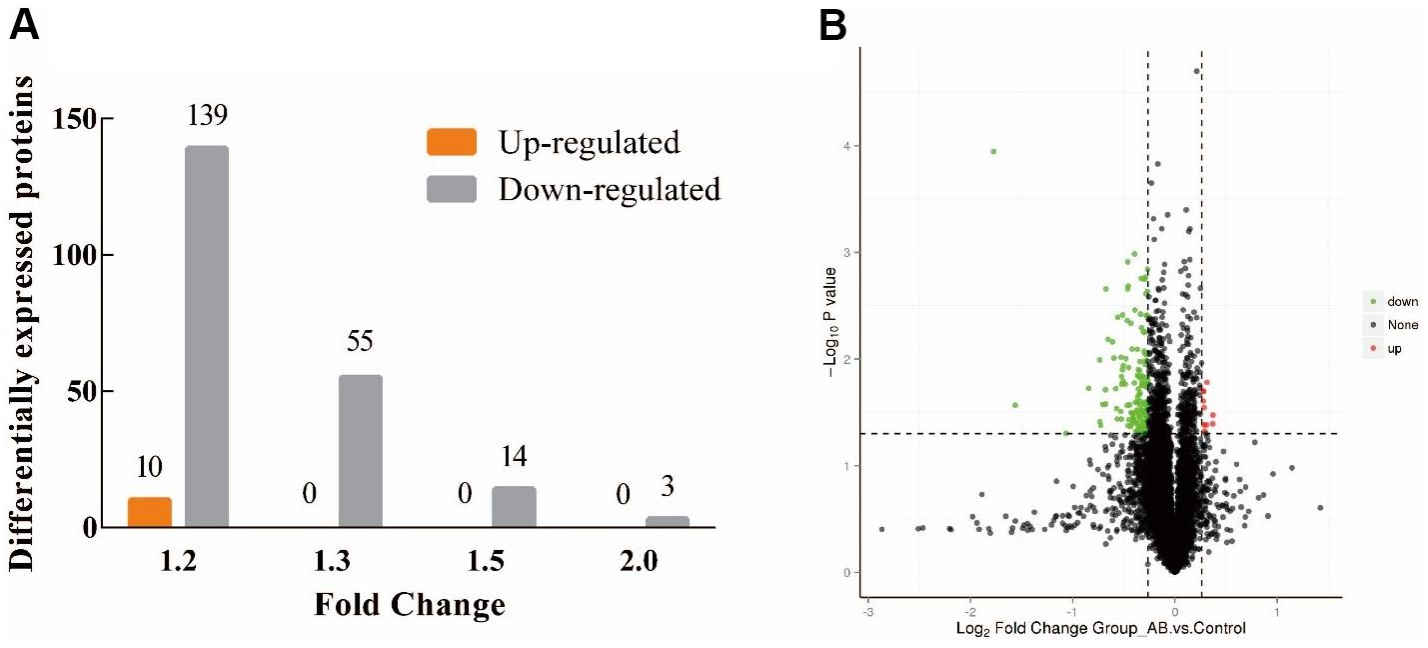
**Distribution of proteins identified in the hypothalamus of KM mice.** (**A**) The amount of up/down-regulated of the DEPs at different FC; (**B**) Volcano plot of the DEPs at FC=1.2. (Red: up-regulated, green: down-regulated).

Figure 3A shows the proteomic results of the hypothalamus of JuA+B high-dose KM group mice compared to the control group. This processing successfully identified 221 DE proteins (6739 proteins in total). The amount of downregulated protein (211) exceeded the amount of upregulated protein [10] in the DE proteins between the two groups, and the upregulated amount in the JuA + B high-dose group was 14 times higher than that in the control group at FC=1.2, (Figure 3B). The volcano plot showed that each treatment group had its own set of upregulated and downregulated proteins (red, upregulated; green, downregulated). All information regarding DEPs is shown in Supplementary Table 1. Therefore, we speculated that the intake of JuA+B significantly downregulated the expression of some proteins in the hypothalamus.

### GO enrichment of differentially quantified proteins

**Figure 4:**
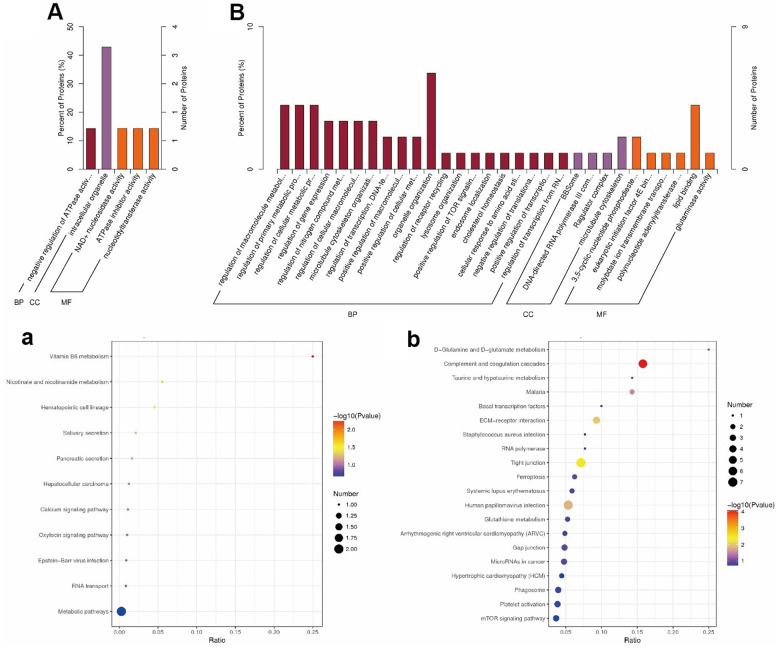
Go (**A**, **B**) and KEGG (**a**, **b**) enrichment results (**A**, **a**) Up-regulated; (**B**, **b**) Down-regulated. In (**A**, **B**), Value of x-axis: $\frac{\text{the number of DEPs in the corresponding pathway}}{\text{the number of total proteins identified in the pathway}}$; The color of the point: the p-value of the hypergeometric test; The size of the point: the number of DE proteins in the corresponding pathway.

We classified the DE proteins into biological process (BP), cellular component (CC), and molecular function (MF) (Supplementary Table 1), according to the GO classification (Figure 4A, 4B). Among the upregulated proteins (Figure 4A), proteins with functions in intracellular organelles (CC) showed the highest percentage. Among the downregulated proteins (Figure 4B), organelle organization (BP) and lipid-binding (MF) were the key processes of these DEPs, suggesting that co-administration of JuA+B could improve sleep through these specific mechanisms.

### KEGG pathways for improvement sleep by JuA+B

To explore the related pathways of JuA+B in the hypothalamus for sleep improvement, a KEGG pathway enrichment analysis was conducted (Figure 4A, 4B). Most DEPs in up-regulated proteins appeared in metabolic pathways (Figure 3A) and the highest degree of protein enrichment was in vitamin B6 metabolism. In the downregulated proteins (Figure 4B), most DE proteins appeared in the complement and coagulation cascades, and vitamin B6 metabolism had the highest level of protein enrichment. Thus, sleep improvement by JuA+B was closely related to these pathways. To find the JuA+B that had the greatest impact, we found five typical enrichment pathways in the process of sleep improvement by JuA+B (*p* < 0.05), as shown in Table 1. The enriched KEGG pathways and proteins with significant changes are also listed, and a description is given in Supplementary Table 1.

**Table 1:** KEGG pathways enrich results (*p* < 0.05).

**Pathway ID**	**Pathway name**	**Protein ID**
04610	Complement and coagulation cascades	Q3V1T9 P29788 Q2I0J8 A2A998 Q5FW62 Q3UER0
04530	Tight junction	Q3UP61 E9PYX7 P39447 Q3UX10 Q8VBX6 Q60771 A0A571BEG7
04512	ECM-receptor interaction	P29788 Q3TR40 Q2I0J8 O54890
05165	Human papillomavirus infection	Q3UP61 P29788 Q3TR40 Q2I0J8 B1AY10 O54890 A0A571BEG7
05144	Malaria	A8DUV3 Q3TR40

Among the five KEGG pathways identified above, tight junction (TJ) was especially noteworthy and also had the most DE proteins: DLG1 (Q3UP61), Afadin (E9PYX7, AF-6), ZO-1(P39447), Tuba3 (Q3UX10, DNMBP), MUPP1 (Q8VBX6, MPDZ), Claudin11 (Q60771, CLDN11), and PATJ (A0A571BEG7, INADL). According to the KEGG database, the expression of these DE proteins affects cell polarity and proliferation, paracellular permeability, actin assembly, tight junction assembly, and cell migration. We believe that JuA+B supplementation affected the expression of key proteins in the TJ, thereby altering the permeability of BBB, which should be considered as the key factor in sleep improvement. Therefore, it is necessary to verify the expression of these DEPs.

### Effect of the JuA+B on the expression of key DE proteins in the hypothalamus

**Figure 5:**
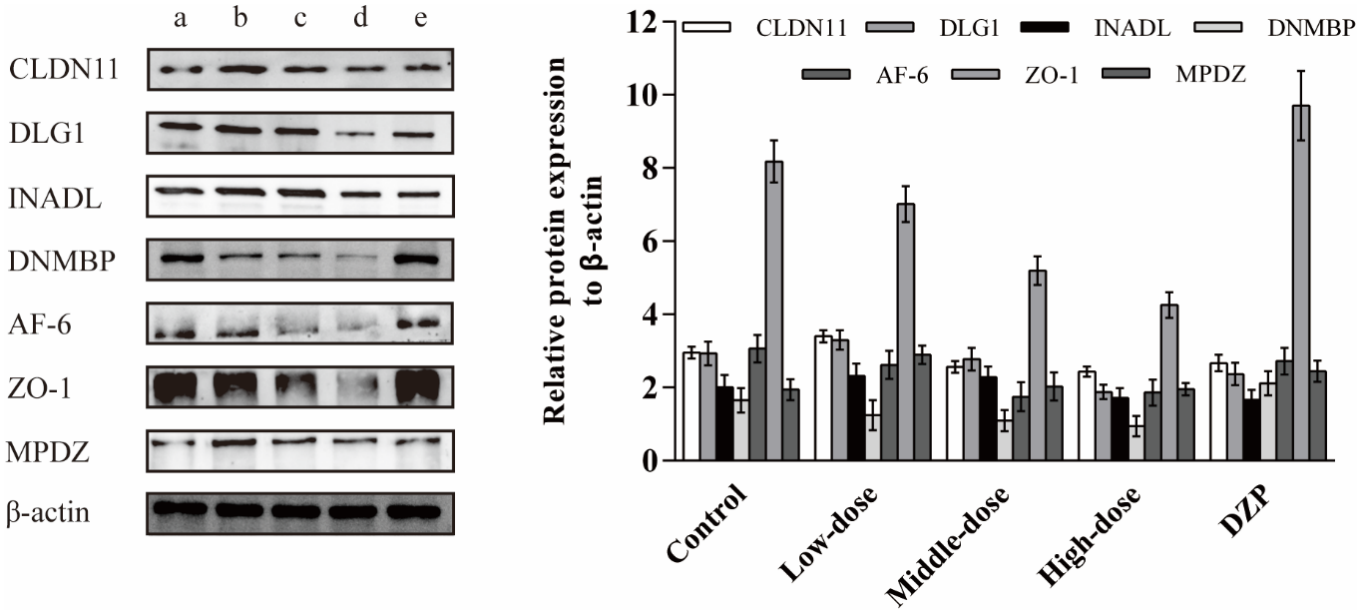
**The effects of the JuA+B on the expression of key DE proteins in the hypothalamus.** (**a**) Control group; (**b**) JuA+B low-dose group; (**c**) JuA+B middle-dose group; (**d**) JuA+B high-dose group; (**e**) DZP group. The protein expression levels of CLDN11, DLG1, INADL, DNMBP, AF-6, ZO-1, and MPDZ were measured by the western blot analysis. The relative intensities of these protein bands were analyzed with ImageJ software. β-actin was used as a control for the protein blots.

To further identify the effect of JuA+B on protein expression in the hypothalamus, the expression levels of the major DE proteins involved in tight junction processing, including CLDN11, DLG1, INADL, DNMBP, AF-6, ZO-1, and MPDZ, were investigated. The expression of all DE proteins decreased with increasing drug dosage (Figure 5), consistent with the proteomics results (Table 1). In addition, compared with the control and DZP groups, the expression of DE proteins in the different dose groups was significantly different; meanwhile, the expression of DE proteins in the control and DZP groups was similar. This showed that the mechanism of Jujuboside and DZP in improving sleep is completely different.

### Potential correlation between the DE proteins

**Figure 6:**
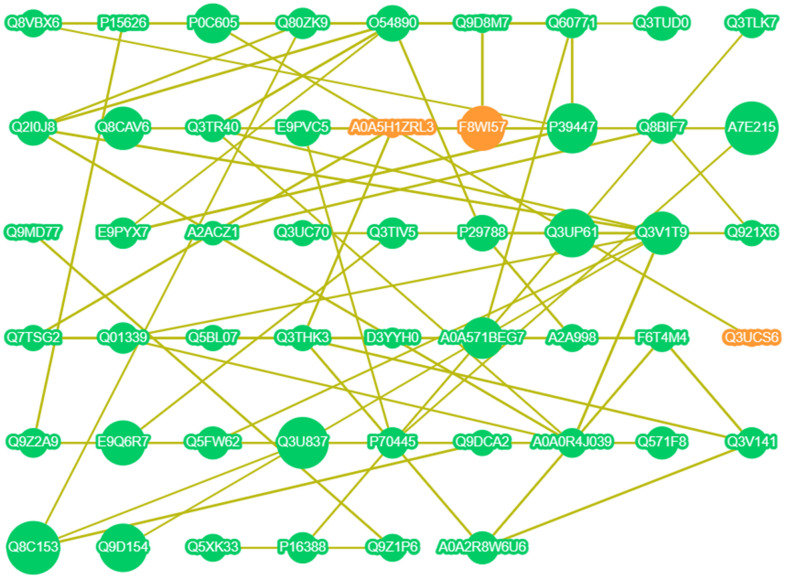
**Interaction network of DE proteins.** Orange point: up-regulated expression; Green point: down-regulated expression; The larger the point, the higher the correlation; The line indicates that there is a correlation.

Figure 6 uses data from the StringDB Protein Interaction Database (http://string-db.org/) and demonstrates the interactions of all DE proteins. Taking the DE protein found in 3.5 as the core, three relationships were observed. The first is the interaction between P39447 (ZO-1), E9PYX7 (Afadin), Q8CAV6, Q8VBX6 (MUPP1), and Q60771 (Claudin11). The second is the interaction between Q8VBX6 (MUPP1), Q60771 (Claudin11), and P39447 (ZO-1). The third is the interaction between Q60771 (Claudin11), Q8VBX6 (MUPP1), P39447 (ZO-1), and A0A571BEG7 (PATJ). These three networks established the interactions between ZO-1, Afadin, MUPP1, Claudin11, and PATJ. Furthermore, ZO-1 may be the source of these interactions. ZO-1 is a high molecular weight polypeptide associated with the TJ in a variety of epithelia [10] and determines the position of claudin polymerization during TJ chain formation [11]. We speculated that the intake of Ju A+B is most likely to initiate a series of effects on the BBB by affecting the expression of ZO-1.

## DISCUSSION

The TJ between endothelial cells is an important part of the blood-brain barrier (BBB) [17], which also affects the body’s sleep state directly [18]. In the present study, the seven DE proteins identified above exhibited a trend of downregulation but with different functions. As a key transmembrane protein in TJ processing [19], Claudin is one of the key upstream proteins in various physiological functions [20]. The down-regulation of Claudin11 could directly lead to the downregulation of ZO-1. In this study, the differential expression of ZO-1 led to direct downregulation of Afadin and Tuba expression. Afadin is a peripheral scaffolding protein similar to ZO-1. Afadin binds to both transmembrane proteins and actin, which are important during junction development and dynamic remodeling [21]. A study on Tuba showed less, but we know that Tuba can influence the tension generated at tricellular corners to change the configuration of cell junctions [22]. PATJ, MUPP1, and DLG1 are downstream proteins in the TJ process. PATJ regulates tight junction formation and polarity in mammalian epithelial cells [23], and MUPP1 shows similar functions and appears in the same PDZ domain as PATJ [24]. The expression of MUPP1 is related to many malignant diseases [25]. Dlg1 is also a tumor suppressor protein [26]. When the expression of Dlg1 is altered, it also affects cell tight junctions [27].

Most of the DE proteins in this study are closely related to sleep or brain health [28–30], and are closely related to the BBB. In addition, some DE proteins are related to neural regulation, including Afadin and Tuba [31, 32]. Therefore, it is speculated that the intake of JuA+B can directly regulate the TJ process in the hypothalamus, thereby interfering with the BBB; its intake may also regulate nerve cells. Therefore, these two functions may exist synergistically. Many other substances from the homology of medicine and food have also been reported to regulate the BBB, including Leonurine [33], and Salvianolic acid [34], which are also active ingredients in traditional Chinese medicine. BBB is also the main pathway for some ingredients, including borneol, to interfere with sleep [35]. In conclusion, we believe that the inference of Ju A + B-assisted sleep in this study is accurate.

The BBB is affected in several ways [36]. As a lipid- soluble component, jujuboside has the potential to enter brain tissue through the BBB. However, based on this study and other reports [2, 37], the intake of JuA+B not only caused the differential expression of related proteins in TJ processing but also interfered with the activity of nerve cells and affected the expression of some neurotransmitters. Therefore, we do not rule out that JuA+B may also produce drug effects by changing the permeability of the BBB indirectly so that other substances can enter brain tissue, which needs further study.

## CONCLUSIONS

This study demonstrated that the simultaneous intake of Jujuboside A and B will affect the levels of key neurotransmitters in serum, protein expression in the hypothalamus, and the related DE protein, which is concentrated in the process of tight junction, reflecting that the intake may have an impact on the blood-brain barrier and its permeability, thereby producing its effect as an aid to sleep. This is not only related to the differential expression of related proteins but also to the expression of neurotransmitters and the activity of neurons. In addition, this study provides a theoretical basis for the modern pharmacological evaluation of jujuboside and the development of functional foods using it as a raw material.

## Supplementary Material

Supplementary Table 1
