# Supplementary material for: The mechanism of simultaneous intake of Jujuboside A and B in the regulation of sleep at the hypothalamic level
Source: Aging (Albany NY). 2023 Sep 5;15(18):9426–37. doi: 10.18632/aging.204995 (PMC10564420; doi:10.18632/aging.204995)
Supplement: Supplementary Table 1 [file aging-15-204995-s001.docx]

**Supplementary Table 1. The information of DE proteins between test groups and control group.**

| Protein Expression | Protein ID | Protein Description |
| --- | --- | --- |
| Up-regulated | Q8K183 | Pyridoxal kinase |
|  | P0C027 | Diphosphoinositol polyphosphate phosphohydrolase 3-alpha |
|  | A0A2R8VHP3 | Predicted pseudogene 5478 |
|  | Q8VEK0 | Cell cycle control protein 50A |
|  | Q3UCS6 | 2-phospho-ADP-ribosyl cyclase |
|  | F8WI57 | Actin-like protein 6B |
|  | Q8K1J6 | CCA tRNA nucleotidyltransferase 1, mitochondrial |
|  | E9PV44 | ATP synthase F1 subunit epsilon |
|  | A0A5H1ZRL3 | Negative elongation factor B |
|  | Q3TNK7 | Coiled-coil domain containing 50 |
| Down-regulated | P97427 | Dihydropyrimidinase-related protein 1 |
|  | Q8C153 | Tr-type G domain-containing protein |
|  | E9PUD2 | Dynamin-1-like protein |
|  | A0A571BDG0 | SRC kinase-signaling inhibitor 1 |
|  | Q3TTX2 | Uncharacterized protein (Fragment) |
|  | Q3UP61 | Uncharacterized protein |
|  | E9PYX7 | Afadin |
|  | E9PVC5 | Eukaryotic translation initiation factor 4 gamma 1 |
|  | A8DUV3 | Alpha-globin |
|  | Q8R317 | Ubiquilin-1 |
|  | Q3U7R1 | Extended synaptotagmin-1 |
|  | Q9Z1P6 | NADH dehydrogenase [ubiquinone] 1 alpha subcomplex subunit 7 |
|  | P39447 | Tight junction protein ZO-1 |
|  | P97315 | Cysteine and glycine-rich protein 1 |
|  | Q9D154 | Leukocyte elastase inhibitor A |
|  | D3YYH0 | PEX5-related protein |
|  | Q922R8 | Protein disulfide-isomerase A6 |
|  | Q3UX10 | Tubulin alpha chain-like 3 |
|  | A2AJI1 | MAP7 domain-containing protein 1 |
|  | Q3TKD0 | Transportin-1 (Fragment) |
|  | Q3U9Q8 | Actin-depolymerizing factor |
|  | A0A0J9YUQ8 | Actin-depolymerizing factor (Fragment) |
|  | A1L0V4 | Histone H3 (Fragment) |
|  | P15626 | Glutathione S-transferase Mu 2 |
|  | S4R1H2 | Ankyrin repeat and sterile alpha motif domain-containing protein 1B |
|  | Q9CSK4 | Uncharacterized protein (Fragment) |
|  | A7E215 | Non-specific serine/threonine protein kinase (Fragment) |
|  | Q3V1T9 | Plasminogen |
|  | E9Q6R7 | Utrophin |
|  | Q8CE04 | Calpain inhibitor |
|  | Q01339 | Beta-2-glycoprotein 1 |
|  | A0A2R8W6U6 | Poly(rC)-binding protein 2 (Fragment) |
|  | E9PX94 | Coiled-coil and C2 domain-containing protein 1A |
|  | A0A0R4J0Z3 | Aquaporin-4 |
|  | A6H663 | BCL2-associated athanogene 3 |
|  | P16388 | Potassium voltage-gated channel subfamily A member 1 |
|  | P97384 | Annexin A11 |
|  | Q3U837 | RNA helicase |
|  | Q14AS7 | Serine (Or cysteine) peptidase inhibitor, clade A, member 3C |
|  | Q3TUD0 | Phosphodiesterase (Fragment) |
|  | Q5EBJ4 | Ermin |
|  | Q8VBX6 | Multiple PDZ domain protein |
|  | Q9CRF7 | Uncharacterized protein (Fragment) |
|  | E9QAF9 | Protein TANC1 |
|  | Q8CII5 | Nucleolysin TIA-1 |
|  | D3YWN7 | Ensconsin |
|  | Q60771 | Claudin-11 |
|  | P29788 | Vitronectin |
|  | A0A7R7YCL1 | GDNF receptor alpha 1e |
|  | Q91WJ7 | SPATS2-like protein |
|  | Q9D0Q7 | 39S ribosomal protein L45, mitochondrial |
|  | Q4FJW2 | Drap1 protein |
|  | Q3TR40 | Uncharacterized protein |
|  | Q8C8D6 | Tr-type G domain-containing protein |
|  | Q6P4T1 | Sorting nexin-19 |
|  | P97298 | Pigment epithelium-derived factor |
|  | Q0VBL3 | RNA-binding protein 15 |
|  | Q9CQ22 | Ragulator complex protein LAMTOR1 |
|  | Q3UZP4 | Small VCP/p97-interacting protein |
|  | A0A0U1RPA0 | Pleckstrin homology domain-containing family A member 7 (Fragment) |
|  | H3BK48 | Melanoma inhibitory activity protein 2 |
|  | P70445 | Eukaryotic translation initiation factor 4E-binding protein 2 |
|  | D3Z4C2 | Stathmin-4 |
|  | Q80ZK9 | WD and tetratricopeptide repeats protein 1 |
|  | Q91W92 | Cdc42 effector protein 1 |
|  | E9PUB0 | Arf-GAP with Rho-GAP domain, ANK repeat and PH domain-containing protein 1 |
|  | D6RFU9 | Synaptophysin-like protein |
|  | P0C605 | cGMP-dependent protein kinase 1 |
|  | Q9DCA2 | 28S ribosomal protein S11, mitochondrial |
|  | Q2I0J8 | von Willebrand factor |
|  | D3Z7X0 | Acyl-Coenzyme A dehydrogenase family, member 12 |
|  | P70455 | Gibbon ape leukemia virus receptor (Fragment) |
|  | H3BJG3 | XK-related protein (Fragment) |
|  | A2A998 | Complement component C8 alpha chain |
|  | Q3TLK7 | Myotubularin |
|  | A2ACZ1 | Probable rRNA-processing protein EBP2 (Fragment) |
|  | Q3THK3 | General transcription factor IIF subunit 1 |
|  | Q923Z3 | Protein MTO1 homolog, mitochondrial |
|  | A2APT9 | Kelch domain-containing protein 7A |
|  | Q5FW62 | Serine (Or cysteine) peptidase inhibitor, clade D, member 1 |
|  | E0CX41 | Leucine-rich repeat transmembrane neuronal protein 1 (Fragment) |
|  | O88502 | High affinity cAMP-specific and IBMX-insensitive 3,5-cyclic phosphodiesterase 8A |
|  | Q571F8 | Glutaminase liver isoform, mitochondrial |
|  | Q6P6L6 | Gem (Nuclear organelle) associated protein 4 |
|  | Q3TTA7 | E3 ubiquitin-protein ligase CBL-B |
|  | Q8C080 | Sorting nexin-16 |
|  | A0A0R4J039 | Histidine-rich glycoprotein |
|  | Q8CAV6 | Protein kinase C |
|  | Q6NSR8 | Probable aminopeptidase NPEPL1 |
|  | Q7TSG2 | RNA polymerase II subunit A C-terminal domain phosphatase |
|  | Q3TIV5 | Zinc finger CCCH domain-containing protein 15 |
|  | F8WGT2 | Transmembrane protein 209 |
|  | Q059K7 | XK-related protein |
|  | F6T4M4 | Serine/arginine repetitive matrix protein 1 (Fragment) |
|  | P40936 | Indolethylamine N-methyltransferase |
|  | Q9Z2A9 | Glutathione hydrolase 5 proenzyme |
|  | Q0HA38 | Tetratricopeptide repeat protein 21B |
|  | Q8VDD9 | PH-interacting protein |
|  | B1AY10 | Transcriptional repressor NF-X1 |
|  | Q673H1 | Tumor suppressor candidate gene 1 protein homolog |
|  | O54890 | Integrin beta-3 |
|  | Q7TN73 | N-acetylneuraminate 9-O-acetyltransferase |
|  | Q6P3Z4 | Zinc finger protein 37 |
|  | Q8BIF7 | WD_REPEATS_REGION domain-containing protein |
|  | Q3UEC0 | Peptidase M12B domain-containing protein (Fragment) |
|  | Q3UC70 | GTP cyclohydrolase 1 |
|  | Q8CH36 | Proton-coupled amino acid transporter 4 |
|  | A0A0R4J0B3 | Serine/threonine-protein kinase |
|  | A2AAU2 | Voltage-dependent calcium channel gamma-4 subunit |
|  | Q9D8M7 | PHD finger protein 10 |
|  | O09113 | Homeobox protein orthopedia |
|  | Q5Y5T2 | Palmitoyltransferase ZDHHC18 |
|  | Q2LEK6 | Embryonic-specific truncated midkine B |
|  | Q544H9 | Uncharacterized protein |
|  | Q3V460 | Gene model 561, (NCBI) |
|  | Q5XPI3 | E3 ubiquitin-protein ligase RNF123 |
|  | A2AUC6 | Bardet-Biedl syndrome 5 protein homolog |
|  | Q921X6 | DNA-directed RNA polymerase III subunit RPC6 |
|  | E9QMD2 | Ubiquitin-conjugating enzyme E2Q-like protein 1 |
|  | Q8K003 | Translation machinery-associated protein 7 |
|  | Q921Y4 | Molybdate-anion transporter |
|  | Q9MD77 | NADH-ubiquinone oxidoreductase chain 4L |
|  | Q3UER0 | Uncharacterized protein |
|  | E5CYJ9 | Protein unc-79 homolog |
|  | A0A0N4SUM3 | Lysophospholipid acyltransferase 5 |
|  | Q8R121 | Protein Z-dependent protease inhibitor |
|  | Q9CR64 | Protein kish-A |
|  | Q5XK33 | Succinate dehydrogenase cytochrome b560 subunit, mitochondrial |
|  | A0A654ICL5 | Connexin p1 |
|  | Q5BL07 | Peroxisome biogenesis factor 1 |
|  | D3YTZ9 | Protein RIC-3 |
|  | Q5XKN4 | Protein jagunal homolog 1 |
|  | A0A0G2JFK4 | Cytochrome c oxidase assembly protein COX19 |
|  | Q8BV79 | TPR and ankyrin repeat-containing protein 1 |
|  | J3QNK5 | Histone deacetylase complex subunit SAP130 |
|  | A0A571BEG7 | InaD-like protein |
|  | A8C756 | Thyroid adenoma-associated protein homolog |
|  | Q9DC04 | Regulator of G-protein signaling 3 |
|  | Q3V141 | Polynucleotide adenylyltransferase (Fragment) |
